# Genome characteristics of clinical *Salmonella enterica* population from a state public health laboratory, New Hampshire, USA, 2017–2020

**DOI:** 10.1186/s12864-022-08769-1

**Published:** 2022-07-23

**Authors:** Madison R. Turcotte, Joshua T. Smith, Jinfeng Li, Xinglu Zhang, Kristin L. Wolfe, Fengxiang Gao, Christopher S. Benton, Cheryl P. Andam

**Affiliations:** 1 University at Albany, State University of New York, Albany, NY USA; 2 Broad Institute of MIT and Harvard, Cambridge, MA USA; 3 New Hampshire Department of Health and Human Services, 29 Hazen Drive, Concord, NH USA

**Keywords:** *Salmonella enterica*, Antimicrobial resistance, Genome, Epidemiology, Evolution

## Abstract

**Background:**

The implementation of whole genome sequencing (WGS) by PulseNet, the molecular subtyping network for foodborne diseases, has transformed surveillance, outbreak detection, and public health laboratory practices in the United States. In 2017, the New Hampshire Public Health Laboratories, a member of PulseNet, commenced the use of WGS in tracking foodborne pathogens across the state. We present some of the initial results of New Hampshire’s initiative to transition to WGS in tracking *Salmonella enterica*, a bacterial pathogen that is responsible for non-typhoidal foodborne infections and enteric fever. We characterize the population structure and evolutionary history of 394 genomes of isolates recovered from human clinical cases in New Hampshire from 2017 to 2020.

**Results:**

The New Hampshire *S. enterica* population is phylogenetically diverse, consisting of 78 sequence types (ST) and 67 serotypes. Six lineages dominate the population: ST 11 serotype Enteritidis, ST 19 Typhimurium, ST 32 Infantis, ST 118 Newport, ST 22 Braenderup, and ST 26 Thompson. Each lineage is derived from long ancestral branches in the phylogeny, suggesting their extended presence in the region and recent clonal expansion. We detected 61 genes associated with resistance to 14 antimicrobial classes. Of these, unique genes of five antimicrobial classes (aminocoumarins, aminoglycosides, fluoroquinolones, nitroimidazoles, and peptides) were detected in all genomes. Rather than a single clone carrying multiple resistance genes expanding in the state, we found multiple lineages carrying different combinations of independently acquired resistance determinants. We estimate the time to the most recent common ancestor of the predominant lineage ST 11 serotype Enteritidis (126 genomes) to be 1965 (95% highest posterior density intervals: 1927–1982). Its population size expanded until 1978, followed by a population decline until 1990. This lineage has been expanding since then. Comparison with genomes from other states reveal lack of geographical clustering indicative of long-distance dissemination.

**Conclusions:**

WGS studies of standing pathogen diversity provide critical insights into the population and evolutionary dynamics of lineages and antimicrobial resistance, which can be translated to effective public health action and decision-making. We highlight the need to strengthen efforts to implement WGS-based surveillance and genomic data analyses in state public health laboratories.

**Supplementary Information:**

The online version contains supplementary material available at 10.1186/s12864-022-08769-1.

## Background

The implementation of whole genome sequencing (WGS) by public health laboratories is a transformative and significant advance in epidemiology, food safety and public health. Established in 1996, PulseNet (National Molecular Subtyping Network for Foodborne Disease Surveillance) is a collaborative effort among state, local, and food regulatory public health laboratories across the United States to quickly detect clusters of disease cases and link potential food and/or environmental sources [1]. PulseNet has transitioned to implementing standardized methods in WGS workflows, from DNA extraction, DNA library preparation and sequencing to data processing and storage across member laboratories. WGS provides superior discriminatory power to characterize genetic variants over the previous method of pulse-field gel electrophoresis (PFGE), and is therefore certainly valuable for disease surveillance, source attribution, and outbreak detection. Genome sequences are publicly shared in real time; hence, data from PulseNet also benefit researchers and scientists who carry out further analyses to understand the biology of foodborne pathogens. In particular, WGS studies of standing pathogen diversity will provide critical insights into the population and evolutionary dynamics that explain the co-circulation of distinct lineages, thus providing a more nuanced picture of the burden of foodborne infections at the local level.

*Salmonella enterica* is a ubiquitous human and animal pathogen that causes substantial economic losses and major public health concerns worldwide [2]. It contains over 2600 recognized serotypes that can be divided into typhoidal and non-typhoidal serotypes, each characterized by unique epidemiological and ecological characteristics [3]. Non-typhoidal *Salmonella* is frequently associated with diarrheal illness or self-limiting gastroenteritis in humans around the world [3, 4]. Most people recover without specific treatment. However, in some cases, particularly in children, elderly and immunocompromised individuals, the associated dehydration can become severe and life-threatening [2]. Global estimates suggest 197 million cases of infection and 84,799 deaths annually from non-typhoidal *S. enterica* in 2016 [5]. Non-typhoidal *S. enterica* can also cause invasive diseases that have higher case fatality than is seen with non-invasive infections [6]. *S. enterica* that cause typhoid and paratyphoid fevers is reported to cause 135,900 deaths globally in 2017 [7].

Antimicrobial resistance remains a serious challenge in the treatment and control of *S. enterica* infections. Multidrug-resistant strains are linked to more severe disease outcomes [8] and can be passed on along the food chain, from production to consumption [9]. In their 2019 Antimicrobial Resistance Report, the Centers for Disease Control and Prevention (CDC) classified antimicrobial resistant typhoidal and non-typhoidal *S. enterica* as serious threats that require systematic surveillance and prompt and sustained action [10].

Here, we aim to characterize the population structure and evolutionary history of 394 *S. enterica* genomes from isolates recovered from human clinical cases in New Hampshire, USA from 2017 to 2020. These genomes were sequenced by the New Hampshire Public Health Laboratories, a member of the PulseNet network. Results show that the local *S. enterica* population is genetically diverse, consisting of multiple co-circulating lineages that have been persisting for years within the state. This study presents some of the initial results of the state’s initiative to implement WGS in public health surveillance of *S. enterica* and the spread of antimicrobial resistance. We highlight some of the major challenges to implementing WGS in state public health laboratories as well as the value of strengthening collaborations between public health officials and genomic scientists.

## Results

### Genomic characteristics and population structure

We compiled a total of 394 *S. enterica* isolates from human clinical cases in New Hampshire, USA (Supplementary Table 1). Of these, 44 isolates were collected in 2017, 158 isolates in 2018, 156 isolates in 2019 and 36 isolates in 2020. The county with the highest number of cases was Hillsborough County, located in the southern part of the state, and which accounted for 26.4% (104/394) of the total isolates.

Across the entire dataset, we identified a total of 21,142 genes that comprise the pan-genome (Supplementary Figure 1 and Supplementary Table 2). Of these, the core genes (present in > = 99% strains) consisted of 3265 genes which represents approximately 15.4% of the entire pan-genome. Together, the core genes and soft-core genes (*n* = 247 genes; present in 95% ≤ strains < 99%) constitutes 16.6% of the pan-genome. The accessory genome is made up of the shell genes (*n* = 1482 genes; present in 15% ≤ strains < 95%) and cloud genes (*n* = 16,148 genes; present in < 15% of strains), which together constitutes 83.4% of the pan-genome. Average nucleotide identity values (ANI) for all pairs of genomes ranged from 94.83 to 100% (Supplementary Table 3).

**Fig. 1:**
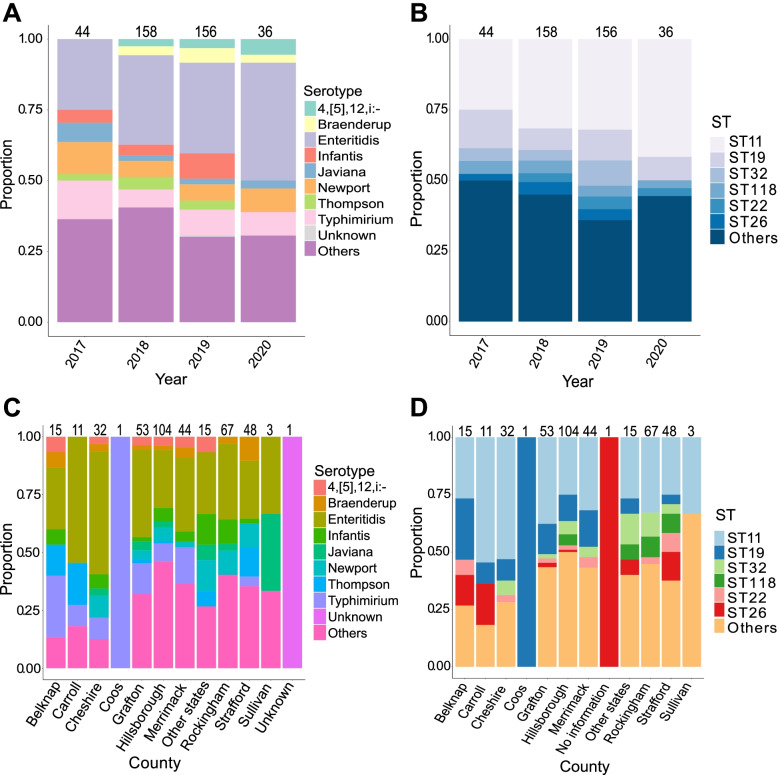
Distribution of serotypes and sequence types (STs) per year (**A**, **B**) and per county (**C**, **D**). Numbers above each bar indicate the total number of genomes. For visual clarity, only the most frequently detected serotypes and STs are shown. Serotype identity shown here is based on the agglutination serotyping method. Full list of serotypes and STs can be found in Supplementary Table 1

Determining the serotypes and sequence types (ST) of isolates are critical to surveillance and source tracking of *S. enterica* because different variants often demonstrate unique phenotypic characteristics [11, 12]. Using in silico analysis of the genome sequences, we classified isolates into 78 STs and 67 serotypes (Supplementary Table 1). Throughout the 4 years of sample collection, the most frequently detected were ST 11 (serotype Enteritidis) and ST 19 (serotype Typhimurium) (Fig. 1AB). Other commonly found serotypes were Braenderup, Infantis, Javiana, Newport, and Thompson, while other commonly found STs were STs 22, 26, 32, and 118. The agglutination method also identified two Typhi and six Paratyphi. Although the number of genomes differed dramatically per county, we found different STs and serotypes that are widely distributed across the 10 counties of the state (Fig. 1CD).

However, we found several inconsistencies in the serotyping results using the agglutination and in silico SeqSero2 methods, with 361/394 or 91.62% of the isolates showing concordance between the two methods (Supplementary Table 1). In all, there were 58 serotypes and one undetermined serotype detected by serology (agglutination), while there were 67 serotypes detected using Seqsero2. There were 11 isolates in 2018 and 22 isolates in 2019 that showed differences between the two serotyping methods.

**Fig. 2:**
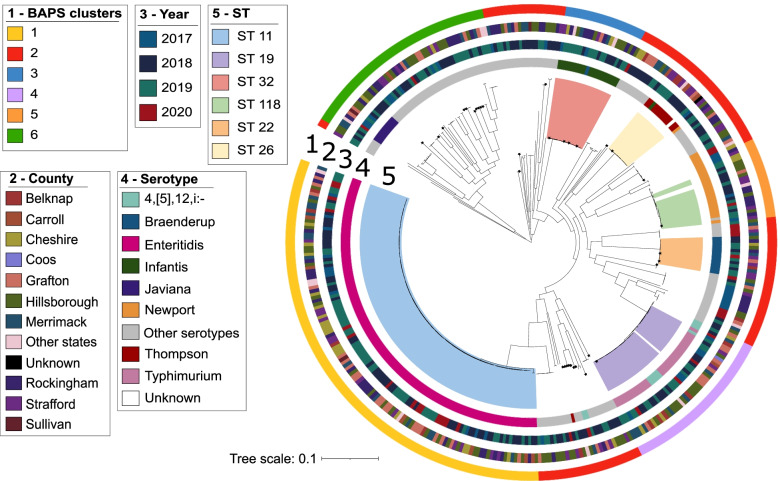
Midpoint-rooted maximum likelihood phylogenetic tree based on 3265 core genes. The scale bar represents the number of nucleotide substitutions per site. Serotype identity shown here is based on the agglutination serotyping method. The black stars on the tip of branches indicate the 33 isolates that had conflicting results from the agglutination test and SeqSero2. BAPS clusters (outermost ring) indicate the sequence clusters determined by RhierBAPS

Population structure analysis using Bayesian hierarchical clustering of the core genome alignment showed six distinct sequence clusters and one cluster encompassing all the sequences that could not be classified with confidence by RhierBAPS (Fig. 2). The six distinct clusters largely corresponded to STs. The largest cluster consisted of isolates classified as ST 11 serotype Enteritidis (*n* = 126/394 or 31.5% of the isolates). Other clusters correspond to the commonly found STs were STs 19 (*n* = 38 genomes), 32 (*n* = 22 genomes), 118 (*n* = 16 genomes), 22 (*n* = 13 genomes), and 26 (*n* = 14 genomes). Each of the six sequence clusters were derived from long ancestral branches in the phylogeny, which suggest their extended presence in the region and recent clonal expansion. The remaining genomes (*n* = 165) made up rare genotypes that can potentially increase in the population with a change in ecological conditions (e.g., change in human demography or antibiotic consumption).

### Multiple resistance genes in diverse genetic backgrounds

**Fig. 3:**
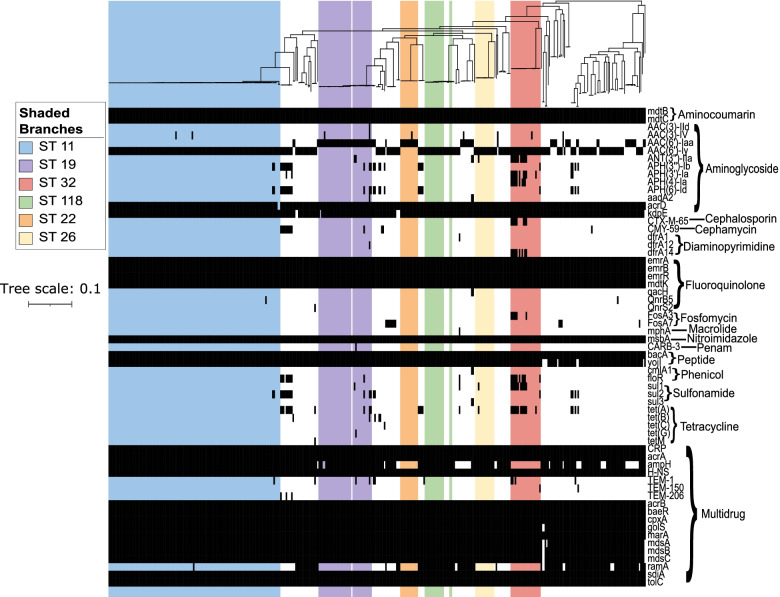
Distribution of antimicrobial resistance genes. Gene presence-absence matrix showing the distribution of antimicrobial resistance genes across the phylogeny (tree is identical to that in Fig. 2). Black blocks indicate presence of gene listed to the right of the panel. The colored columns represent the STs. Names of the antimicrobial classes are indicated on the right of the resistance genes

We used ABRicate to determine the presence of acquired antimicrobial resistance genes in our dataset (Fig. 2 and Supplementary Table 4). We identified a total of 61 unique genes associated with resistance to 14 different antimicrobial classes (aminocoumarins, aminoglycosides, cephalosporins, cephamycins, diaminopyrimidines, fluoroquinolones, fosfomycins, macrolides, nitroimidazoles, penams, peptides, phenicols, sulfonamides, tetracyclines). Of these, unique genes of five antimicrobials (aminocoumarins, aminoglycosides, fluoroquinolones, nitroimidazoles, and peptides) were detected in all genomes, with each genome carrying at least one resistance gene associated with each antimicrobial class. All genomes carried > 10 unique resistance gene (Fig. 3 and Supplementary Figure 2). The 18 most common resistance genes were *cpxA*, *CRP, acrA, H-NS*, *acrB, acrD, bacA, baeR, emrA, emrB, emrR, marA, mdtB, mdtC, mdtK, msbA, sdiA* and *tolC*. Genes conferring resistance to multiple antimicrobials were also prevalent and included *acrA, acrB, baeR, cpxA, CRP, H-NS, marA, sdiA* and *tolC* (Fig. 3, Supplementary Figure 2 and Supplementary Table 5).

### Clonal origin and population dynamics

**Fig. 4:**
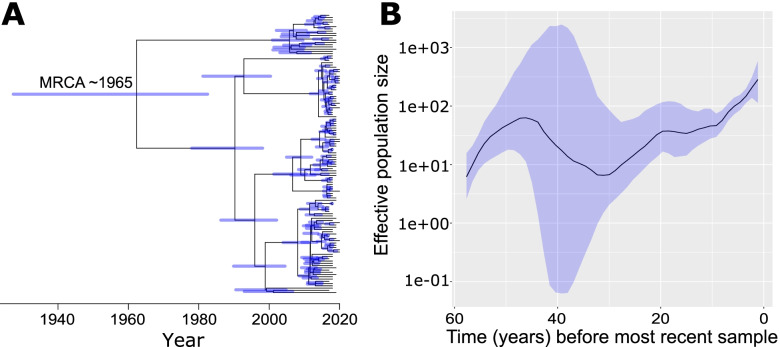
Bayesian phylogeny and population dynamics of sequence cluster 1 (Enteritidis ST 11; *n* = 126 genomes). **A** Bayesian maximum clade credibility time-calibrated phylogeny based on non-recombining regions of the core genome. Blue bars indicate 95% confidence intervals. **B** Bayesian skygrowth plot that depicts changes in effective population size over time. Median is represented by a black line and 95% confidence intervals are in blue

To provide a historical perspective on the New Hampshire *S. enterica* population, we constructed a time-calibrated phylogeny using the core genome alignment. Here, we focused only on the largest sequence cluster (Enteritidis ST 11) which enabled us to obtain sufficient amount of genetic variation to estimate a molecular clock. We observed a slight but significant positive correlation between the dates of isolation and root-to-tip distances (R^2^ = 0.03 and *p* = 2.11^− 02^) (Supplementary Figures 3 and 4), indicating the presence of a clock-like signal. We estimated the time to the most recent common ancestor (tMRCA) of this sequence cluster to be 1965 (95% highest posterior density intervals: 1927–1982) (Fig. 4A). For this sequence cluster, we also estimated the change in the effective population size (Fig. 4B), which is a measure of the rate of change in population composition due to genetic drift [46]. Results indicate that its population size expanded until 1978 after its initial emergence, followed by a population decline until 1990. This sequence cluster has been expanding since then.

### Relationship of New Hampshire isolates with the broader United States population

**Fig. 5:**
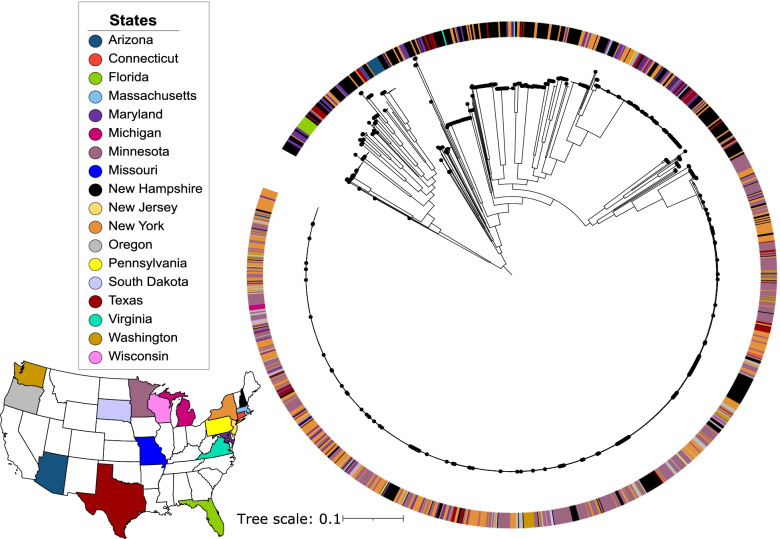
Midpoint-rooted maximum likelihood phylogenetic tree of 1354 *S. enterica* genomes from the United States based on the alignment of 225,784 core SNPs. The scale bar represents the number of nucleotide substitutions per site. The black dots indicate the New Hampshire genomes. Full list of strain names, accession numbers and associated metadata can be found in Supplementary Table 6. The colors in the outer ring of the tree represent the 18 states from where the genomes came from, which also correspond to the colors on the map

We next sought to place the genetic diversity of the New Hampshire *S. enterica* isolates within the broader United States population. We used a genome data set consisting of 960 clinical *S. enterica* isolates from the Pathogen Detection database of the National Center for Biotechnology Information (NCBI) (Supplementary Table 6). These genomes represented 17 other states in the country. In all, this larger dataset consisted of 1354 genomes. We generated a maximum likelihood tree using the alignment of 225,784 single nucleotide polymorphisms (SNPs) from core genes of the entire dataset (Fig. 5). Results showed that the New Hampshire genomes were intermingled with those from other states across the phylogeny, even among very closely related strains. The lack of clustering of isolates according to their state of origin reflects close relationship and widespread mobility of geographically disparate isolates.

## Discussion

WGS technology is critical to public health surveillance efforts and responsive food safety measures at the state and local levels. The state of New Hampshire has recently transitioned to WGS and our study presents the initial results of sequencing *S. enterica* from clinical cases sampled from 2017 to 2020. Here, we also wanted to show that beyond the standard strain-level characterization, we can also infer relevant information about the antimicrobial resistance, population and evolutionary dynamics of the pathogen that will be valuable to understanding foodborne diseases in local communities.

Our study shows that *S. enterica* isolates harboring multiple resistance genes did not originate from the geographical expansion of a single clone; instead, a variety of resistance determinants in different combinations are carried by multiple phylogenetically distinct lineages. One of these predominant lineages (ST 11 serotype Enteritidis) has been circulating in New Hampshire for more than five decades, with evidence of sustained increase in the population. Hence, the evolution of *S. enterica* in New Hampshire is shaped mainly by both the long-term co-circulation of the six dominant lineages and the multiple independent acquisitions of resistance genes. These processes can lead to new, emerging lineages that can rapidly spread across the state and beyond over relatively short timeframes. A large suite of rarer genotypes is equally problematic as their population dynamics can change due to alterations in antimicrobial use, food safety practices, land use patterns (e.g., agricultural intensification, changes in animal husbandry), trade, and interactions at the wildlife–livestock–human interface. These rare genotypes also act as an important reservoir of new variants of resistance genes that the more dominant lineages can acquire through horizontal gene transfer [13, 14]. Monitoring of both common and rare genotypes over the long-term and comparison between states can help inform public health and regulatory decision-making actions. As we have shown in this study, additional analyses beyond strain characterization (e.g., time-calibrated phylogeny, effective population size) are important to understand the historical local context of foodborne pathogens and to infer the underlying causes of either persistence or replacement of STs and serotypes over time. Moreover, across the United States, antimicrobial use varies considerably between and within states [15]. Such variation will inevitably influence the selection for certain *S. enterica* lineages and the prevalence of specific resistance genes. Additionally, the intermingled phylogenetic relationship of genomes from different states means that long-distance dissemination of genotypes with clinically relevant characteristics such as multidrug resistance can rapidly spread beyond state borders. Such WGS analyses are essential in state public health laboratories to provide historical and geographical contexts to understand the origins of locally spreading genotypes.

Our study highlights major challenges to the full implementation of WGS for foodborne outbreak response and surveillance at the New Hampshire Public Health Laboratories, which is likely true for any state public health agency across the country. Foremost is the need for creating an information technology infrastructure for genome data analyses and developing bioinformatics expertise at the state level. Bioinformatics analyses can be carried out using in-house pipelines, web and/or cloud-based computational tools, and outsourcing to collaborators or other laboratories [16]. The use of the first two options is limited by strict institutional requirements and availability of appropriate operating systems. Intersectoral collaboration between public health and food safety authorities (e.g., GenomeTrackr) will also strengthen bioinformatics analyses and investigations of outbreaks and surveillance activities of foodborne pathogens [17]. Here, we show that collaborations with universities and academic research laboratories can also be an effective approach to supporting state public health laboratories by providing computing power, bioinformatics expertise, and software. To ensure that WGS results are interpretable and actionable within a useful timeframe, there must be clear and continued communication between the state public health laboratory and university researchers. If WGS surveillance is to have a real-world impact on disease outbreak detection and management that is rapid and timely, it is imperative that WGS data analyses must be closely incorporated into state public health laboratories. Long-term support and investment in appropriately trained staff (e.g., bioinformaticians) and computational resources for WGS data analyses in state public health laboratories are critical.

Second, procurement of pertinent epidemiological information and other kinds of descriptive data associated with sequenced isolates presents another challenge. At present, the New Hampshire Public Health Laboratories rely on the receipt of bacterial isolates or samples from clinical laboratories. Hence, certain regions within the state may be over-represented while others remain invisible to current surveillance strategies. For example, in our study, there were many more isolates received from certain counties (Hillsborough, Rockingham, Grafton) than from others, which may lead to misinterpretation of results. Sequence data must also be carefully interpreted alongside epidemiological and laboratory data associated with each isolate, while maintaining patient privacy. If information from these foodborne pathogens is not included (e.g., food consumption activities, clinical symptoms, treatment outcomes, travel history), the sensitivity of disease cluster detection, transmission patterns and source attribution is reduced. Unfortunately, these data were not available to us. Integrating comprehensive and standardized epidemiological information for each isolate, as well as contact tracing and human demographic data, with genomic sequencing will enable a variety of investigations relevant to public health: tracking and reconstructing spatial scales of transmission, identification and isolation of superspreading events, distinguishing repeated introductions versus continuing local spread, differentiating outbreaks due to clonal expansion versus multiple co-circulating independent transmission chains, temporal and spatial scales at which interventions are most impactful, forecasting the likely spread of a pathogen from within households and hospitals to regional and global scales, and predicting the severity of disease outcomes and populations at risk. Coupling genomic and non-genomic information will have a formidable positive impact on effectively deploying rapid, responsive, and real-time actions by public health laboratories.

Third, we found some inconsistencies between the agglutination and in silico methods of serotyping, with 33/394 (or 8.38%) of the isolates having varying results from the two methods. Similar conflicting results have been reported in previous studies comparing traditional serotyping methods and WGS. For example, out of 1041 *S. enterica* isolates analyzed by the US Food and Drug Administration, SeqSero assignments differed from traditional serological testing in 80 isolates (7.7% of the total) and no serotype prediction was determined from 62 isolates (5.9%) [18]. This is lower than that reported in another study of 520 clinical isolates, whereby SeqSero exhibited 98% concordance with traditional serotyping, but monophasic variants were often misindentified [19]. SeqSero is also known to be unable to predict the O-7 antigen [19]. Fine-tuning of genome-based antigenic determination is therefore necessary for reliable detection of specific and rarer serotypes. Another possible source of discrepancy is that traditional serotyping methods may potentially lead to false positive results due to weak or non-specific agglutination [20], particularly when distinguishing closely related serovars and polyphyletic serovars [21, 22]. Moreover, unidentified serotypes can result from autoagglutination and loss of antigen expression [21, 22]. These ambiguous cases therefore require additional serological testing and more specific antisera.

## Conclusions

Strengthening efforts to fully implement WGS-based surveillance and data analyses in state public health laboratories is critical. WGS studies of standing pathogen diversity will provide critical insights into the population and evolutionary dynamics of distinct pathogen lineages and antimicrobial resistance, which can be translated to effective public health action and decision-making.

## Methods

### Sample collection

Bacterial isolates were submitted to the Public Health Laboratories, New Hampshire Department of Health and Human Services (DHHS), Concord, New Hampshire, USA from 2017 to 2020. When New Hampshire first implemented WGS in 2017, only those *S. enterica* isolates that were specifically requested by state epidemiologists at the Bureau of Infectious Disease Control (BIDC) or the Centers for Disease Control and Prevention (CDC) were sequenced. Beginning in 2018, all *S. enterica* isolates received by the Public Health Laboratories were sequenced. Our dataset also included isolates received only during the first half of 2020. Isolates were received from health care providers across the state of New Hampshire and were collected from patients who were diagnosed with *Salmonella* infection. Isolates were recovered mostly from stool, with a few isolates from bile, blood, and urine samples. In the state of New Hampshire, *Salmonella* infections must be reported to the DHHS within 72 hours of a suspected or confirmed case. If there is a suspected case of typhoidal *Salmonella* infection, this must be reported within 24 hours. Isolate submission to DHHS is not mandatory but highly encouraged. There were 15 isolates that were obtained from patients who came from neighboring states, but who were diagnosed in New Hampshire. These were also included in our analysis as it was unknown whether they were infected while they were in New Hampshire. No identifiable information is associated with the isolates. In total, our initial dataset included 458 *S. enterica* isolates. Serotype was determined at the New Hampshire Public Health Laboratories by agglutination of the bacterium with specific antisera to identify variants of the two surface structures O and H antigens based on the White-Kauffmann-Le Minor (WKL) scheme [23].

### DNA extraction and whole genome sequencing

Sequencing of *S. enterica* isolates is part of the nationwide surveillance program PulseNet, a United States national laboratory network that connects foodborne illness cases to detect outbreaks and is sponsored by the Centers for Disease Control and Prevention (CDC) [1]. New Hampshire Public Health Laboratories is a PulseNet participating laboratory. We used Pulsenet’s standard operating procedures (https://www.cdc.gov/pulsenet/index.html) to carry out DNA extraction, library preparation and whole genome sequencing. Briefly, DNA extraction procedures were conducted using the DNeasy Blood & Tissue Kit (Qiagen, Valencia CA). DNA quality and concentration were measured using Qubit fluorometer and NanoDrop spectrophotometer. A total of 1 ng of genomic DNA from each isolate was used to construct sequencing libraries using the Nextera XT DNA Library Preparation Kit (Illumina, Inc. San Diego, CA) following the manufacturer’s instructions. Samples were sequenced as multiplexed libraries on the Illumina MiSeq platform operated per the manufacturer’s instructions for 500 cycles to produce paired end reads of 250 bp in length. Raw reads of all *S. enterica* genomic sequences generated under PulseNet USA surveillance [24] are uploaded in real-time to the sequence read archive (SRA) hosted by NCBI. Accession numbers are listed in Supplementary Table 1.

### De novo genome assembly and annotation

We used the assembly pipeline program Shovill v.1.1.0 (https://github.com/tseemann/shovill) with the --trim option to yield high-quality genomes. Shovill implements a series of steps to improve assemblies, including read subsampling to a reasonable depth of 150x, read error correction, trimming adaptor sequences, detecting and removing sequencing errors, and assembling using SPAdes [25]. Genome quality was assessed for all assemblies using QUAST v.5.0.2 [26] and CheckM v.1.1.3 [27] with cutoff thresholds of > 200 contigs and < 40,000 base N50 as exclusion criteria. We also excluded those genomes which are < 90% complete and have > 5% contamination. Our final dataset used for all downstream analyses consisted of 394 genomes. Genomes were annotated using Prokka v.1.14.5 [28]. We used fastANI v.1.32 [29] with a 95% threshold to confirm species identity.

### Pan-genome, phylogenetic and clustering analyses

The entirety of genes present in the dataset or pan-genome [30] was assessed using Roary v.3.11.2 with default settings [31]. Roary iteratively clusters protein sequences using CD-HIT [32], all-against-all BLASTP [33] and Markov clustering [34]. Nucleotide sequences were aligned using MAFFT v.7.477 [35]. The core genome determined by Roary was used as input in SNP-sites v.2.5.1 [36] to identify SNPs. The core genome SNP alignment was then used to build a maximum likelihood phylogenetic tree using the program RAxML v.8.2.12 [37] with the general time-reversible (GTR) model of nucleotide substitution and Gamma model of rate heterogeneity. The phylogenetic tree was then visualized using the Interactive Tree of Life [38]. We partitioned the strains into sequence clusters consisting of genetically similar individuals using the Bayesian hierarchical clustering algorithm RhierBAPS v.1.1.3 [39].

To place the New Hampshire in the broader *S. enterica* population in the United States, we examined the 307,733 clinical *S. enterica* genomes available in the Pathogen Detection database hosted by NCBI (https://www.ncbi.nlm.nih.gov/pathogens/) as of May 31, 2022. From these, we narrowed down the U.S. clinical isolates to include only those sequences from human samples and that had information about the state of origin. This yielded a total of 3019 genomes. Due to computational resource limitations, we had to reduce the number of genomes for analyses and therefore randomly selected 966 genomes. These genomes were filtered further using CheckM [27] to remove any genomes that were > 5% contaminated or < 90% completed. We compared the 966 genomes to a *S. enterica* reference genome (RefSeq assembly accession ID: GCF_000006945.2) using fastANI [29] to ensure species identity. Our final non-New Hampshire U.S. dataset consisted of 960 genomes representing 17 states. Using Snippy v4.6.0 (https://github.com/tseemann/snippy), a total of 225,784 core SNPs were identified, aligned, and mapped to the reference genome (RefSeq assembly accession ID: GCF_000006945.2). The core SNPs were extracted and used to build a phylogenetic tree using FastTree v2.1.10 [40] using the GTR model of nucleotide substitution.

### In silico sequence typing, serotyping and antimicrobial resistance detection

ST of each strain was determined using MLST v.2.19.0 (https://github.com/tseemann/mlst), a program which extracts seven single-copy housekeeping genes (*aroC, dnaN, hemD, hisD, purE, sucA, thrA*) and compares their sequence identity to previously deposited allele combinations in the *S. enterica* PubMLST database (https://pubmlst.org/organisms/salmonella-spp) [41]. In addition to the conventional laboratory serotyping method described above, we also used the k-mer-based algorithm SeqSero2 to predict the serotype based on the sequences of the O and H antigens [42]. To detect horizontally acquired antimicrobial resistance genes, we used ABRicate v.1.0.0 (https://github.com/tseemann/abricate) using threshold values of > 95% sequence identity and > 95% sequence coverage to known resistance genes deposited in the Comprehensive Antibiotic Resistance Database [43].

### Temporal structure and population demography

Using a recombination-free core genome phylogeny generated by Gubbins [44], we used BactDating v.1.1, a Bayesian method for estimating the molecular clock rate and coalescent rate [45]. We first determined whether there was sufficient genetic change between sampling times to reconstruct a statistical relationship between genetic divergence and time. We carried out a root-to-tip linear regression analysis and calculation of the coefficient of determination (R^2^). When a significant positive correlation between the dates of isolation and root-to-tip divergence was observed, we inferred the dates when common ancestors are estimated to have existed [45]. We used a mixed clock model and 10^7^ iterations to conduct molecular dating of the nodes of the tree. We removed the first half of iterations as burn-in and subsequently sampled every 100 iterations. We used Skygrowth v.0.3.1 to estimate the changes in effective population size over time [46].

## Supplementary Information

**Additional file 1: Supplementary Table 1.** Accession numbers, sampling information, MLST profiles and genome characteristics of the 394 *S. enterica*. Serotyping results by agglutination test and SeqSero2 that were not in concordance are highlighted in yellow. **Supplementary Table 2.** Pan-genome characteristics determined using Roary. **Supplementary Table 3.** Matrix of pairwise ANI values calculated using fastANI. **Supplementary Table 4.** Presence and absence of antimicrobial resistance genes per genome identified using ABRicate to query the Comprehensive Antibiotic Resistance Database. **Supplementary Table 5.** List of genes conferring resistance to multiple antimicrobials identified using ABRicate to query the Comprehensive Antibiotic Resistance Database. **Supplementary Table 6.** Strain names, accession numbers and associated metadata of 960 genomes from 17 other states in the United States. These genomes were obtained from the NCBI Pathogen Detection database.

**Additional file 2: Supplemental Figure 1.** Pan-genome characteristics determined using Roary. Pie chart showing the classification of the genes in the pan-genome: core genes (genes present in > = 99% strains), soft-core genes (genes present in 95% ≤ strains < 99%), shell genes (genes present in 15% ≤ strains < 95%), and cloud genes (genes present in < 15% of strains). **Supplemental Figure 2.** Distribution of antimicrobial resistance genes. (Top) Histogram showing the distribution of the number of antimicrobial resistance genes per genome. Only genes conferring resistance to a single antimicrobial compound are included here. (Bottom) Histogram showing the distribution of the number of genomes carrying genes conferring resistance to multiple antimicrobial compounds. **Supplemental Figure 3.** Bactdating statistical tests for sequence cluster 1 (Enteritidis ST 11). (Left) Initial rooted phylogeny. X-axis represents the number of single nucleotide polymorphisms. (Right) correlation test between date and root-to-tip distance within the phylogeny. Color of dots correspond to year of sampling: Blue – 2017, dark purple – 2018, light purple – 2019, red – 2020. **Supplemental Figure 4.** Bactdating trace plots for sequence cluster 1 (Enteritidis ST 11) constructed by periodic sampling over the MCMC runs.

## Data Availability

The dataset supporting the conclusions of this article is included within the article and its additional files. Genome sequence data of the New Hampshire *S. enterica* isolates have been deposited in the NCBI Sequence Read Archive under BioProject accession number PRJNA230403 with BioSample accession numbers for each genome listed in Supplementary Table 1. Accession numbers and associated data of genomes from other states are publicly available in NCBI Pathogen Detection database and are listed in Supplementary Table 6.

## Abbreviations

WGS: Whole genome sequencing
ST: Sequence type
MLST: Multilocus sequence typing
tMRCA: Time to the most recent common ancestor
