# Supplementary material for: Genome characteristics of clinical Salmonella enterica population from a state public health laboratory, New Hampshire, USA, 2017–2020
Source: BMC Genomics. 2022 Jul 23;23:537. doi: 10.1186/s12864-022-08769-1 (PMC9308939; doi:10.1186/s12864-022-08769-1)
Supplement: Supplementary file 2 — Additional file 2: Supplemental Figure 1. Pan-genome characteristics determined using Roary. Pie chart showing the classification of the genes in the pan-genome: core genes (genes present in > = 99% strains), soft-core genes (genes present in 95% ≤ strains < 99%), shell genes (genes present in 15% ≤ strains < 95%), and cloud genes (genes present in < 15% of strains). Supplemental Figure 2. Distribution of antimicrobial resistance genes. (Top) Histogram showing the distribution of the number of antimicrobial resistance genes per genome. Only genes conferring resistance to a single antimicrobial compound are included here. (Bottom) Histogram showing the distribution of the number of genomes carrying genes conferring resistance to multiple antimicrobial compounds. Supplemental Figure 3. Bactdating statistical tests for sequence cluster 1 (Enteritidis ST 11). (Left) Initial rooted phylogeny. X-axis represents the number of single nucleotide polymorphisms. (Right) correlation test between date and root-to-tip distance within the phylogeny. Color of dots correspond to year of sampling: Blue – 2017, dark purple – 2018, light purple – 2019, red – 2020. Supplemental Figure 4. Bactdating trace plots for sequence cluster 1 (Enteritidis ST 11) constructed by periodic sampling over the MCMC runs. [file 12864_2022_8769_MOESM2_ESM.pdf]

Supplementary Figures

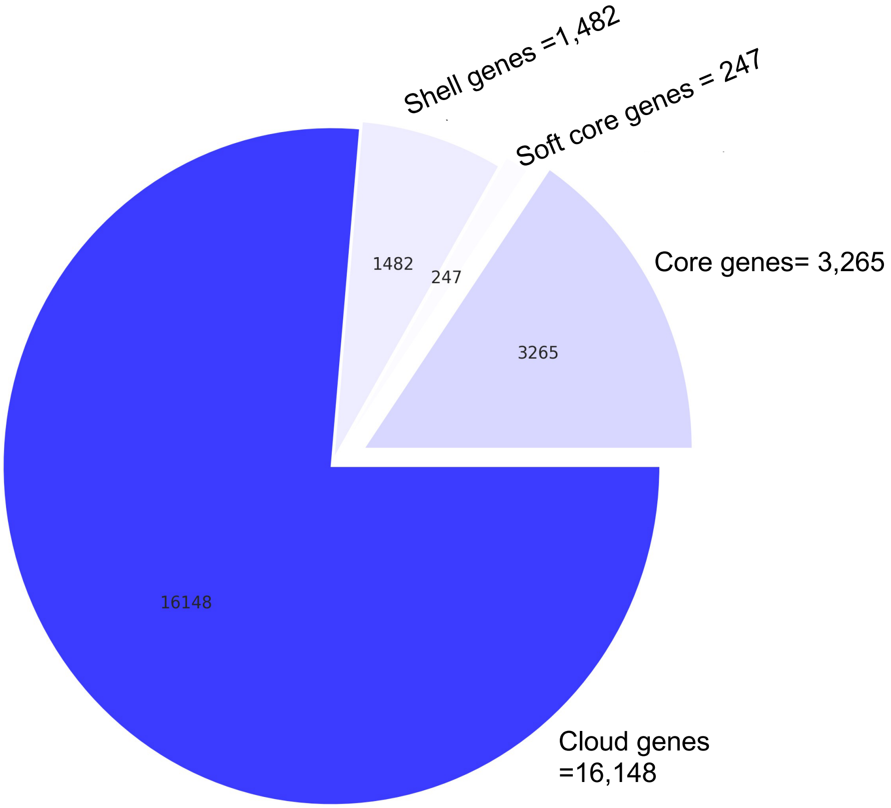

Supplemental Figure 1. Pan-genome characteristics determined using Roary. Pie chart showing the classification of the genes in the pan-genome: core genes (genes present in >=99% strains), soft-core genes (genes present in 95% ≤ strains <99%), shell genes (genes present in 15% ≤ strains <95%), and cloud genes (genes present in <15% of strains)

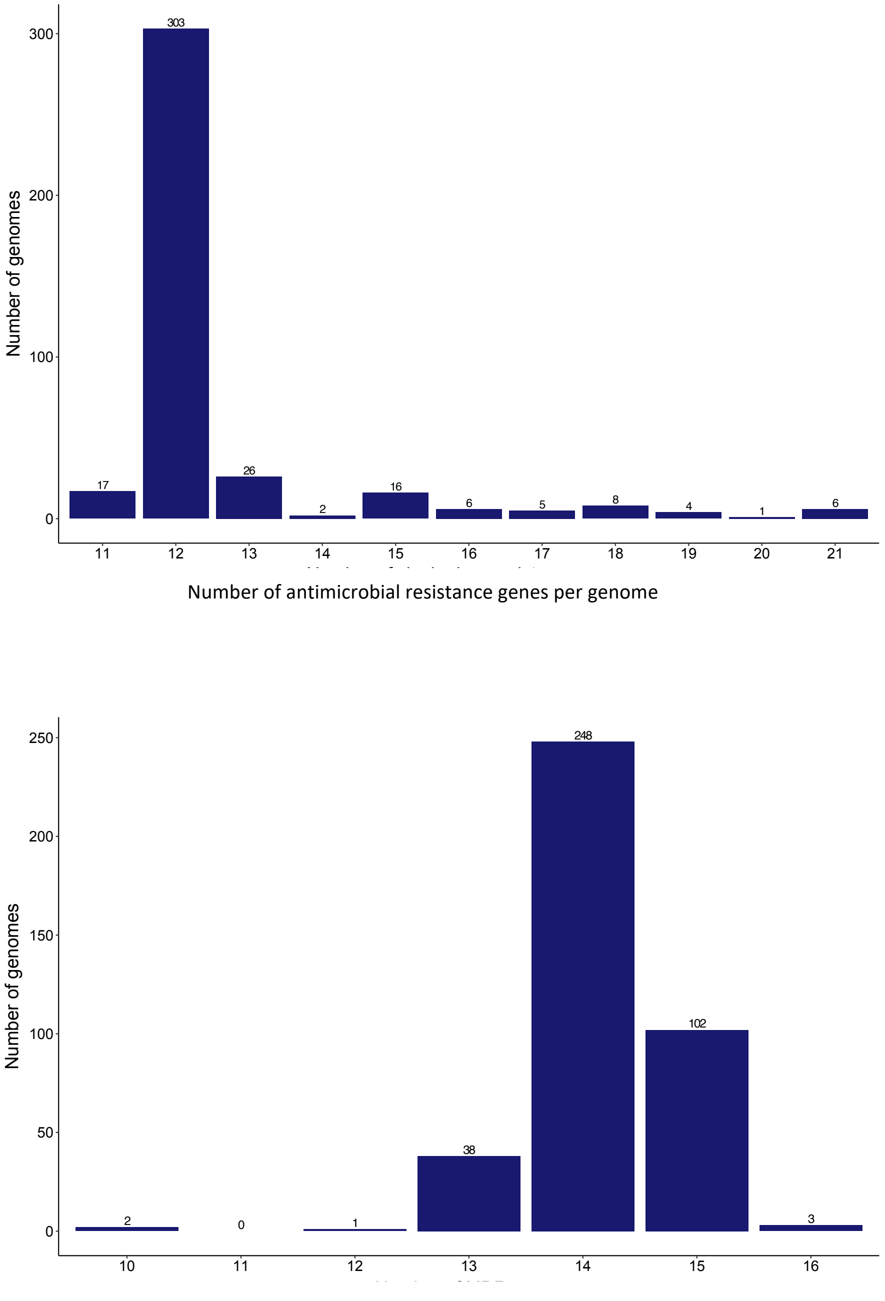

Supplemental Figure 2. Distribution of antimicrobial resistance genes. (Top) Histogram showing the distribution of the number of antimicrobial resistance genes per genome. Only genes conferring resistance to a single antimicrobial compound are included here. (Bottom) Histogram showing the distribution of the number of genomes carrying genes conferring resistance to multiple antimicrobial compounds.

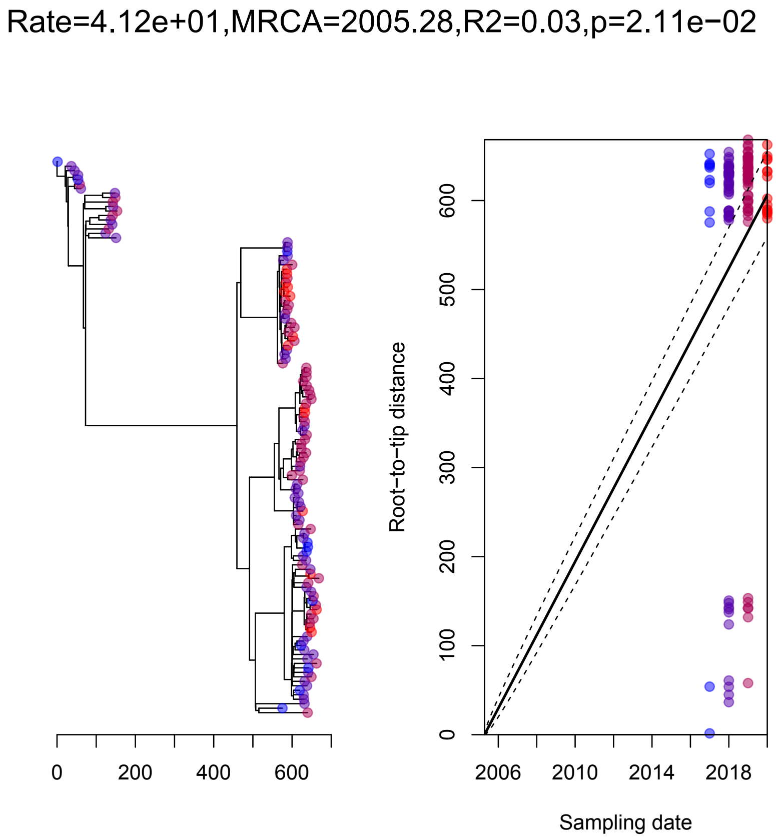

Supplemental Figure 3. Bactdating statistical tests for sequence cluster 1 (Enteritidis ST 11). (Left) Initial rooted phylogeny. X-axis represents the number of single nucleotide polymorphisms. (Right) correlation test between date and root-to-tip distance within the phylogeny. Color of dots correspond to year of sampling: Blue – 2017, dark purple – 2018, light purple – 2019, red – 2020.

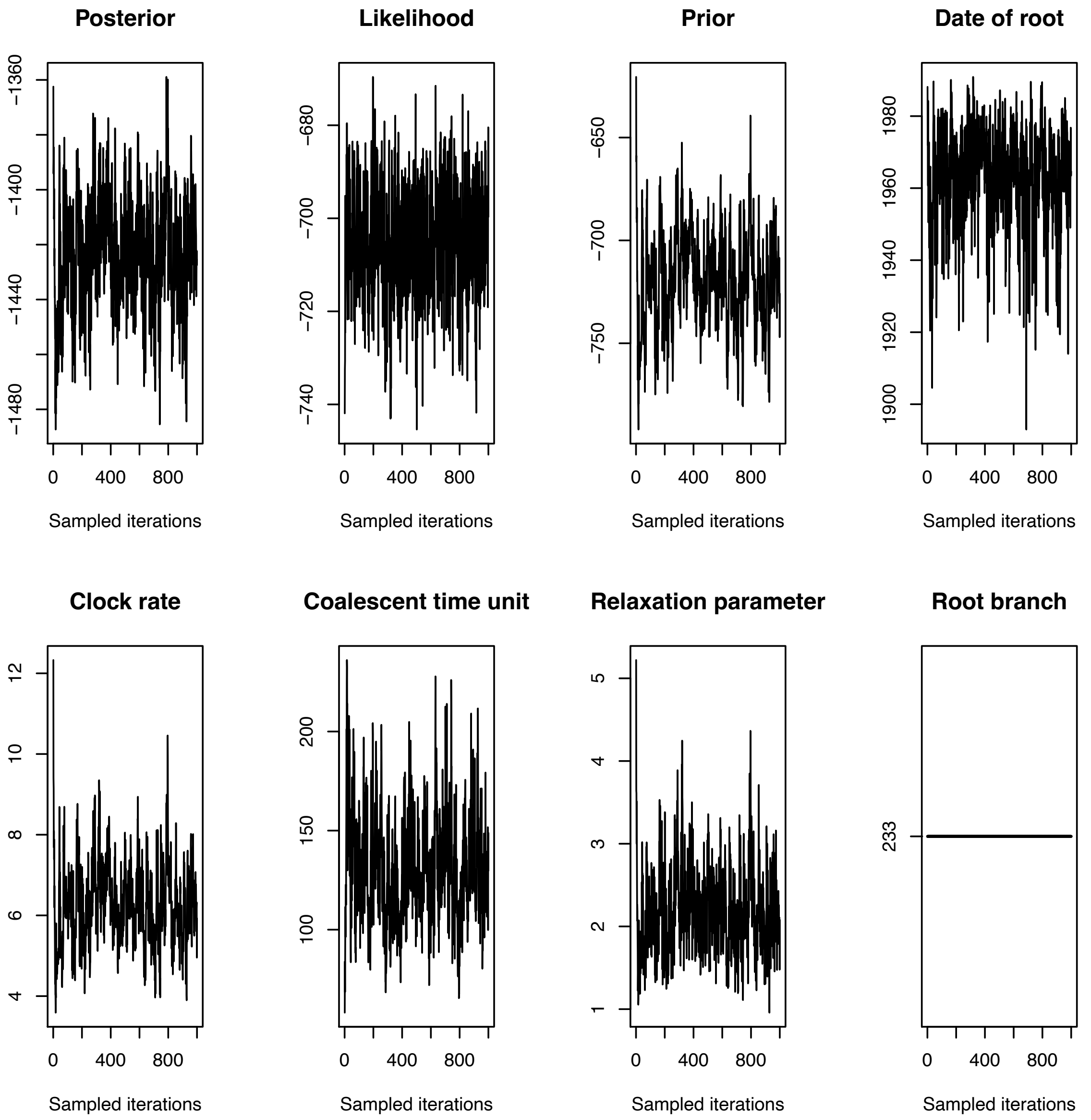

Supplemental Figure 4. Bactdating trace plots for sequence cluster 1 (Enteritidis ST 11) constructed by periodic sampling over the MCMC runs.
